# Nitrogen recycling at the Costa Rican subduction zone: The role of incoming plate structure

**DOI:** 10.1038/s41598-017-14287-y

**Published:** 2017-10-24

**Authors:** Hyunwoo Lee, Tobias P. Fischer, J. Maarten de Moor, Zachary D. Sharp, Naoto Takahata, Yuji Sano

**Affiliations:** 1 Department of Earth and Planetary Sciences, University of New Mexico, Albuquerque, NM 87131 USA; 2 Atmosphere and Ocean Research Institute, The University of Tokyo, Kashiwa, Chiba 277-8564 Japan; 3 Observatorio Vulcanológico y Sismológico de Costa Rica, Universidad Nacional de Costa Rica, Heredia, Costa Rica

## Abstract

Efficient recycling of subducted sedimentary nitrogen (N) back to the atmosphere through arc volcanism has been advocated for the Central America margin while at other locations mass balance considerations and N contents of high pressure metamorphic rocks imply massive addition of subducted N to the mantle and past the zones of arc magma generation. Here, we report new results of N isotope compositions with gas chemistry and noble gas compositions of forearc and arc front springs in Costa Rica to show that the structure of the incoming plate has a profound effect on the extent of N subduction into the mantle. N isotope compositions of emitted arc gases (9–11 N°) imply less subducted pelagic sediment contribution compared to farther north. The N isotope compositions (δ^15^N = −4.4 to 1.6‰) of forearc springs at 9–11 N° are consistent with previously reported values in volcanic centers (δ^15^N = −3.0 to 1.9‰). We advocate that subduction erosion enhanced by abundant seamount subduction at 9–11 N° introduces overlying forearc crustal materials into the Costa Rican subduction zone, releasing fluids with lighter N isotope signatures. This process supports the recycling of heavier N into the deep mantle in this section of the Central America margin.

## Introduction

**Figure 1:**
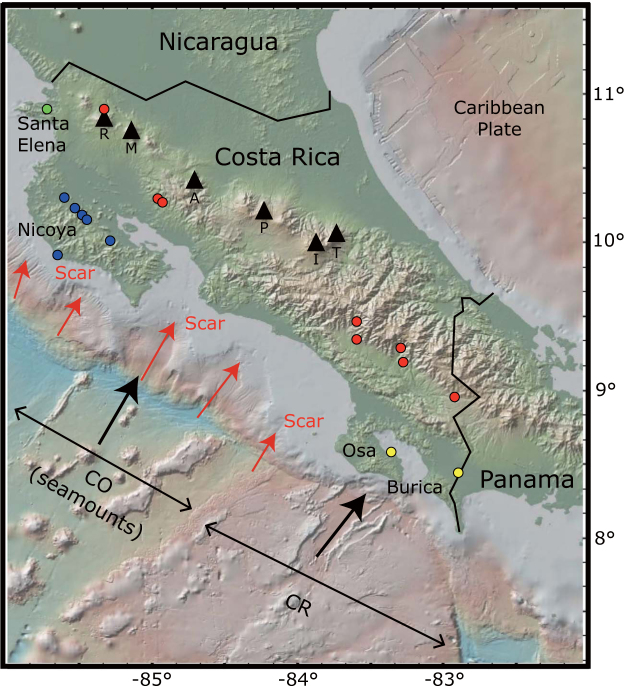
Map of the Costa Rican subduction zone. Sampled forearc (blue and yellow circles) and arc front (red circles) springs are shown. This map was created using GeoMapApp 3.6.4 (http://www.geomapapp.org). Volcanic centers (black triangles) are displayed: R (Rincon de la Vieja), M (Miravalles), A (Arenal), P (Poas), I (Irazu), T (Turrialba). The Cocos plate (CO) with seamounts and the Cocos Ridge (CR) are two major segments in the Costa Rican subduction zone. The subducting plate with seamounts and related scars at the frontal arc are shown at 9–11 N°.

Subduction-zone fluids play a pivotal role in magma generation processes in arc settings. The release of fluids and volatiles from subducting slabs causes melting of the overlying mantle to produce arc magmas^1,2^. Mass balance relationships of geochemical processes have been used to understand subduction processes, recycling of chemical components, mantle heterogeneity, and climate effects^3,4^. As the Costa Rican subduction zone, a part of the Central American margin, has geochemical accessibilities to drilled oceanic samples, forearc fluid seeps, and volcanism on the arc front^3,5–9^ (Fig. 1), this area is an appropriated area to test geochemical mass balance relationships.

Nitrogen (N), the most abundant gas component in air, is one of the major volatiles released by volcanism and hydrothermal activity to the atmosphere^10^. In subduction systems, N in magmatic volatiles reflects pelagic sediment input (δ^15^N = +7‰, ref.^11^) that is subducted with oceanic plates^3,12–14^. In the Central American margin, previously reported N isotope compositions of fumarole and hot spring gas discharges in Guatemala and Nicaragua show such a sediment contribution with δ^15^N values up to 6.3‰ ^3,12^. However, δ^15^N values of fumarole and hot spring gas samples in Costa Rica have been reported with a range from −3.0 to 1.7‰, suggesting more mantle N contribution (δ^15^N = −5‰, ref.^11^). This shows a lower fraction of sediment contribution compared to localities farther north^3,6^. Recycling efficiency of N in the Costa Rican subduction zone is low due to the small N outflux at the arc front compared to the N influx at the trench^6^. This observation has been attributed to off-scraping of sediments or forearc devolatilization of N at the Costa Rica subduction zone^6^. In other regions, a low efficiency of N recycling has been documented in the Sangihe Arc^15^ and in the Mariana Arc^16^. In these locations, sediment off-scraping or lack of organic sediment availability have been invoked as plausible causes for the comparatively low sedimentary N flux out of these volcanic arcs. In order to further constrain the notion of sediment off-scraping or underplating, trenchward regions, such as the forearc, are potential locations where such processes may be observed geochemically. In these areas, devolatilization of pelagic sediments could occur and potentially be sampled in associated springs and groundwaters. However, to date, N isotope compositions of forearc regions have not been measured rendering it impossible to fully constrain the nitrogen cycle at subduction zones.

In this work, we explore forearc regions with new results of N isotope compositions, gas chemistry, and helium isotopes of springs. Costa Rica is the ideal location to perform such a study because in contrast to most subduction zones the forearc is subaerial and accessible to sampling at Santa Elena, Nicoya, Osa, and Burica peninsulas (Fig. 1), where a number of springs are releasing volatiles. We also report new data from springs in the Costa Rica arc front.

## Results

### Gas chemistry

**Figure 2:**
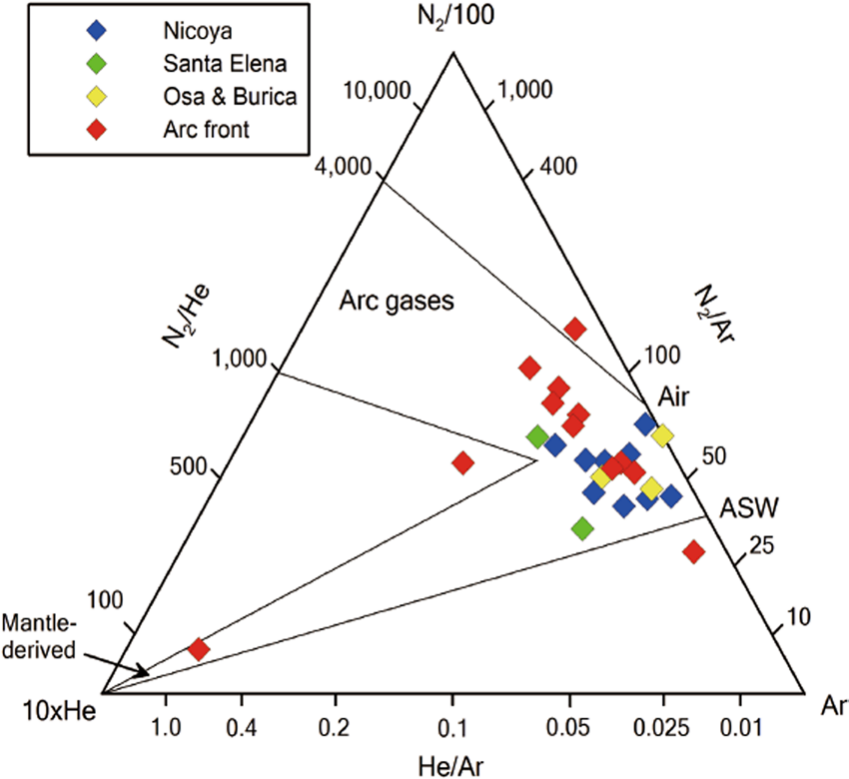
Ternary plot of N_2_-Ar-He abundances. Relative abundances of N_2_, Ar, and He in the forearc and arc front springs samples are used to show mixing relationships among the mantle, arc gases, air, and air saturated water (ASW). The forearc springs plot closer to the air-ASW components than the arc front springs which likely have either the mantle of arc gas end-members.

Forearc (T = 26.0–31.6 °C; pH = 7.0–11.1) and arc front springs (T = 26.1–72.6 °C; pH = 6.5–9.6) in Costa Rica were sampled in 2012 and 2014 (Fig. 1; Table 1). Based on gas compositions, forearc springs are subdivided into N_2_-rich (62.4–98.3 vol. %) and CH_4_-rich (36.1–96.8 vol. %) types, and arc front springs are subdivided into N_2_-rich (88.0–98.5 vol. %) and CO_2_-rich (47.2–98.4 vol. %) types (Supplementary Information). Although air contamination during sampling is minor based on low O_2_ contents (<2.3 vol. %) except for CR12-15, CR14-03, CR14-09B, CR12-05, and CR12-13(6.0–10.3 vol. %) (Supplementary Information), N_2_/Ar ratios for forearc (47–95) and arc front (30–146) springs are similar or slightly higher than ratios of air saturated water (ASW, 40) and air (83) (Table 2). N_2_/He and He/Ar ratios are higher than 1,000 and lower than 0.1, respectively, except for Cayuco (N_2_/He = 84; He/Ar = 0.8) which seems to have more mantle-derived volatiles (Table 2). In Fig. 2, the N_2_-Ar-He abundances show that volatiles in the Costa Rican springs are mostly atmospheric, except for two arc front springs (Cayuco and Rincon de la Vieja) with a higher proportion of mantle-derived components. Given that N_2_/Ar ratios are higher than ASW, N in excess of ASW (N_2-exc_) can be calculated. Ar contents are used to calculate N_2-exc_ values based on the assumption that Ar in volcanic gases and geothermal fluids are mostly from ASW^17,18^. Using measured N_2_ and Ar contents and the N_2_/Ar ratio of ASW (40), N_2-exc_ values are obtained as following^19^:

1 $${{\rm{N}}}_{2-{\rm{exc}}}={{\rm{N}}}_{2}({\rm{measured}})-{40}^{\ast }{\rm{Ar}}({\rm{measured}})$$

**Table 1:** Locations of the sampled Costa Rican springs.

Area		ID	Latitude (°N)	Longitude (°W)	T	pH
**Forearc springs**						
Nicoya	La Conchita Pool	CR14-01	10.47419	85.60569	27.8	7.5
	Rancho El Salitral	CR12-16	10.23211	85.53158	29.6	10.1
		CR14-02			30.5	10.2
	Sabana Grande	CR12-15	10.17906	85.48014	31.5	8.5
		CR14-03			31.6	9.4
	Playa Garza	CR14-06A	9.90792	85.65025	29.8	7.0
		CR14-06B				
	Salitral Vigia	CR14-07	10.10683	85.28606	26.0	8.1
	Salitral San Martin	CR12-14	10.16039	85.46006	29.7	8.5
		CR14-08				
Santa Elena	Rio Murcielago 1	CR14-09	10.89064	85.72600	28.6	11.1
		CR14-09B				
Osa	Sandalo	CR12-01	8.57533	83.36383	29.5	
		CR12-02				
Burica	Laurel	CR12-03	8.44128	82.90483		8.3
		CR12-04				8.1
**Arc front springs**						
Aguas Calientes		CR12-05	8.94697	82.91911	37.2	6.5
		CR12-06				6.4
Yheri		CR12-07	9.19483	83.28081	26.1	7.7
		CR12-08				6.7
Rocas Calientes		CR12-09	9.30289	83.29789	62.8	7.4
		CR12-10				7.8
Montecarlo		CR12-11	9.34400	83.59542	32.3	7.8
		CR12-12				
Aguas Termales Gevi		CR12-13	9.47222	83.60464	36.8	8.1
Pueblo Antiguo		CR12-18	10.28328	84.92925	45.3	7.8
Cayuco		CR12-19	10.28747	84.95564	72.6	6.5
Rincon de la Vieja		CR14-11	10.89772	85.32656	61.5	6.5

**Table 2:** Gas chemistry of N_2_-Ar-He and isotope compositions of the Costa Rican springs.

ID	N_2_/Ar	N_2_/He	He/Ar	N_2exc_/He	δ^15^N-N_2_	±	R/Ra	±	^4^He/^20^Ne
**Forearc springs**									
CR14-01							1.02	0.01	0.40
CR12-16	47	8,721	0.005	1,297	−0.7	0.1			
CR14-02	58	2,195	0.026	676	−2.1	<0.1	0.88	0.01	0.49
CR12-15	66	5,998	0.011	2,377	−1.1	<0.1			
CR14-03	49	4,267	0.011	773	−0.7	<0.1	0.61	0.01	1.37
CR14-06A	68	3,536	0.019	1,453	−2.8	0.2	0.72	0.01	5.74
CR14-06B	72	2,832	0.025	1,260					
CR14-07	49	2,647	0.019	493	−1.4	<0.1	0.98	0.01	0.39
CR12-14	75	25,548	0.003	11,865	−0.9	<0.1			
CR14-08	86		0.036	1,291			0.62	0.01	0.68
CR14-09	46	1,377	0.034	190	0.0	<0.1	1.06	0.01	0.40
CR14-09B	95	2,230	0.043	1,295			1.09	0.12	1.48
CR12-01	51	5,533	0.009	1,221	4.7	0.1			
CR12-02	73								
CR12-03	62	2,848	0.022	1,022	4.0	1.6			
CR12-04	68	785,392	0.0001	320,225					
**Arc front springs**									
CR12-05	65	4,463	0.015	1,707	−0.5	0.1			
CR12-06	59	4,980	0.012	1,609	−0.4	0.1			
CR12-07	143	3,728	0.038	2,687	1.6	<0.1			
CR12-08	116	4,285	0.027	2,808	−0.4	<0.1			
CR12-09	109	3,450	0.032	2,188	−0.2	0.1			
CR12-10	95	4,211	0.022	2,429	0.8	0.1			
CR12-11	91	3,476	0.026	1,941	−0.4	0.1			
CR12-12	64	3,618	0.018	1,356	0.1	0.3			
CR12-13	30	4,722	0.006		−0.8	0.1			
CR12-18	146	13,554	0.011	9,852.996	1.5	<0.1			
CR12-19	68	84	0.804	34	−4.4	1.5			
CR14-11	108	1,179	0.092	743	−0.2	<0.1	7.88	0.18	38.81

Calculated N_2-exc_ contents and N_2-exc_/He ratios indicate N amounts in excess to what is supplied to the sampled water phase by N_2_ derived from air and then dissolved water (Table 2).

### Nitrogen isotope compositions

**Figure 3:**
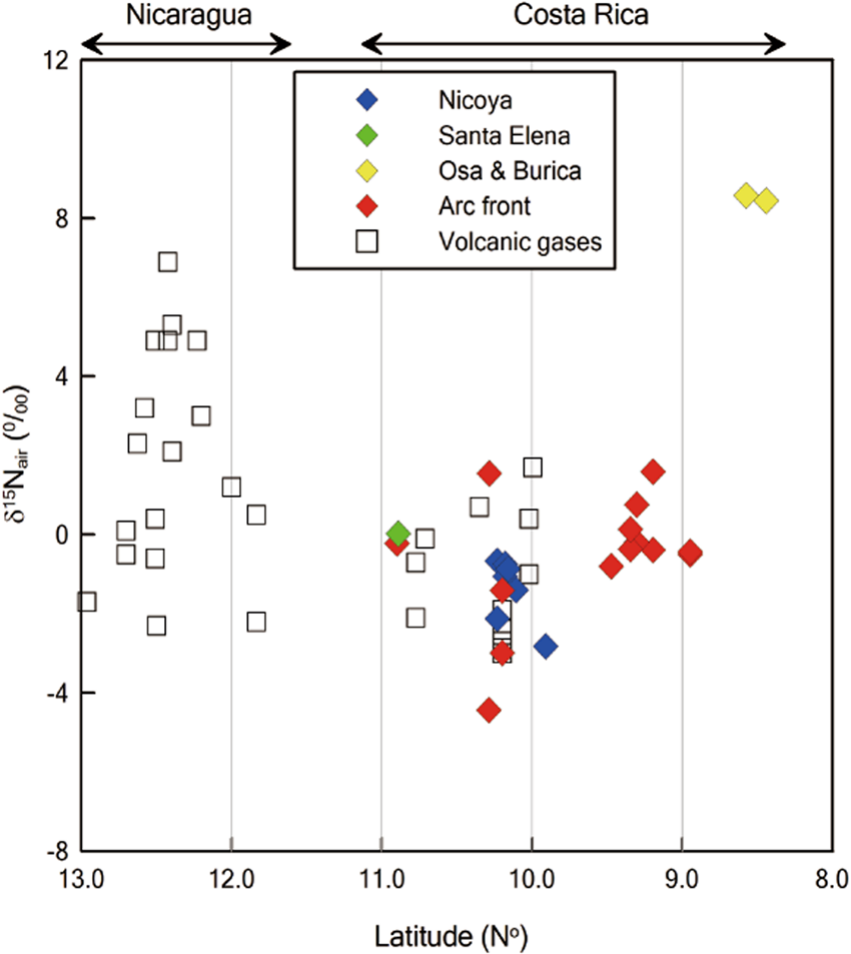
Plot of nitrogen isotope compositions versus latitude. Nitrogen isotope compositions of the Costa Rican forearc and art front springs are displayed together with previously reported volcanic gases in Costa Rica^3,6^ and Nicaragua^12^.

**Figure 4:**
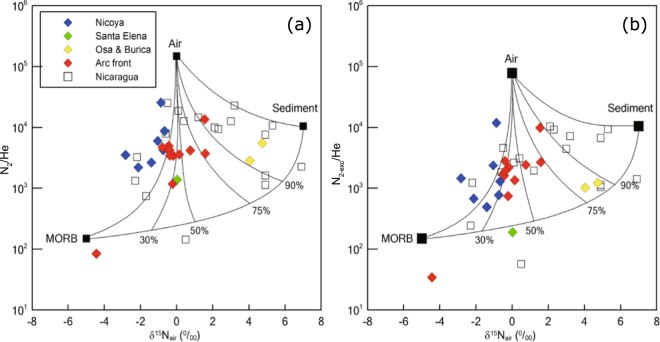
Nitrogen provenance diagram. (**a**) Nitrogen isotopes compositions versus N_2_/He ratios of the Costa Rican springs. End-members and mixing lines are defined as described in refs^3,11^. Percentages of sediment input are shown. The Nicaraguan data is from ref.^12^. (**b**) Nitrogen isotope compositions versus N_2-exc_/He, which displays a less number of data points (for both this study and ref.^12^) off the mix line between MORB and air. N_2-exc_/He of the air end-member was taken by reducing the ASW contribution.

Nitrogen isotope compositions (δ^15^N vs air) of the Costa Rican springs range from −4.4 to 1.6‰ (9–11 N°), except for the Osa (4.7‰) and Burica (4.0‰) forearc springs located in the southernmost part of Costa Rica (8.4–8.6 N°) (Table 2; Fig. 3). The δ^15^N values of the springs are well consistent with the reported volcanic δ^15^N values (−3.0 to 1.9‰, refs^3,6,20^). In Fig. 3, both the forearc and arc front springs at 9–11 N° have less sediment (δ^15^N = 7‰) contribution than other Central American subduction zone samples (δ^15^N = −2.2 to 6.3‰) at > 11 N° (e.g., Nicaragua and Guatemala)^3,12^. The N sources are constrained following the approach of refs^3,11^ by using δ^15^N and N_2_/He ratios of the springs (Fig. 4a):

2 $${{\rm{\delta }}}^{15}{{\rm{N}}}_{{\rm{measured}}}={{\rm{\delta }}}^{15}{{\rm{N}}}_{{\rm{MORB}}}\times {{\rm{f}}}_{{\rm{MORB}}}+{{\rm{\delta }}}^{15}{{\rm{N}}}_{{\rm{sediment}}}\times {{\rm{f}}}_{{\rm{sediment}}}+{{\rm{\delta }}}^{15}{{\rm{N}}}_{{\rm{air}}}\times {{\rm{f}}}_{{\rm{air}}}$$

3 $$1/{({{\rm{N}}}_{2}/{\rm{He}})}_{{\rm{measured}}}={{\rm{f}}}_{{\rm{MORB}}}/{({{\rm{N}}}_{2}/{\rm{He}})}_{{\rm{MORB}}}+{{\rm{f}}}_{{\rm{sediment}}}/{({{\rm{N}}}_{2}/{\rm{He}})}_{{\rm{sediment}}}+{{\rm{f}}}_{{\rm{air}}}/{({{\rm{N}}}_{{\rm{2}}}/\mathrm{He})}_{{\rm{air}}}$$

4 $${{\rm{f}}}_{{\rm{MORB}}}+{{\rm{f}}}_{{\rm{sediment}}}+{{\rm{f}}}_{{\rm{air}}}=1$$

where f_MORB_, f_sediment_, and f_air_ are fractions of three end-members (Mid Ocean Ridge Basalt (MORB), sediment, and air). δ^15^N_MORB_, δ^15^N_sediment_, and δ^15^N_air_, are −5‰, 7‰, and 0‰, and (N_2_/He)_MORB_, (N_2_/He)_sediment_, and (N_2_/He)_air_ are 150, 10,500, and 148,900, respectively^3,11,21–25^. However, δ^15^N values (−2.8 to −0.7‰) of the Nicoya forearc springs and the arc front springs, which have been reported for the Sangihe and Nicaraguan arc systems^15,16^, are shifted towards values more negative than defined by the MORB-air mixing lines (Fig. 4a). In order to account for such negative values, kinetic fractionation processes related to gas bubbling through spring water^26^ and thermal decomposition of ammonia^27^ have been proposed, however, the fractionations associated with these processes (<1‰) are insufficient to explain the measured N isotope shift (Fig. 4a). For these reasons, N sources are constrained using the modified approach with N_2-exc_/He ratios following:

5 $$1/{({{\rm{N}}}_{2-{\rm{exc}}}/{\rm{He}})}_{{\rm{measured}}}={{\rm{f}}}_{{\rm{MORB}}}/{({{\rm{N}}}_{2}/{\rm{He}})}_{{\rm{MORB}}}+{{\rm{f}}}_{{\rm{sediment}}}/{({{\rm{N}}}_{2}/{\rm{He}})}_{{\rm{sediment}}}+{{\rm{f}}}_{{\rm{air}}}/{({{\rm{N}}}_{2-{\rm{exc}}}/{\rm{He}})}_{{\rm{air}}}$$

where (N_2-exc_/He)_air_ is 78,023 by reducing the ASW proportion, and (N_2_/He)_MORB_ and (N_2_/He)_sediment_ are the same values as above because we do not expect any air-derived N in these end-members. In Fig. 4b. the δ^15^N and N_2-exc_/He of the Costa Rican springs and Nicaraguan gases^12^ are displayed showing that now most samples lie within the mixing curves. The air end-member in Fig. 4b now represents N_2_ addition from air in the atmosphere and not dissolved in the water phase. Sediment contribution (f_sediment_) to N of all Costa Rican springs ranges from 0 to 42%, except for the Osa (76%) and Burica (68%) springs (Supplementary Information). Additionally, most of the Costa Rican springs at 9–11 N° shows less sediment contribution compared to Guatemala (f_sediment_ = 20–90%, ref.^3^) and Nicaragua (f_sediment_ = 46–96%, ref.^12^) (Supplementary Information).

### Noble gas geochemistry

**Figure 5:**
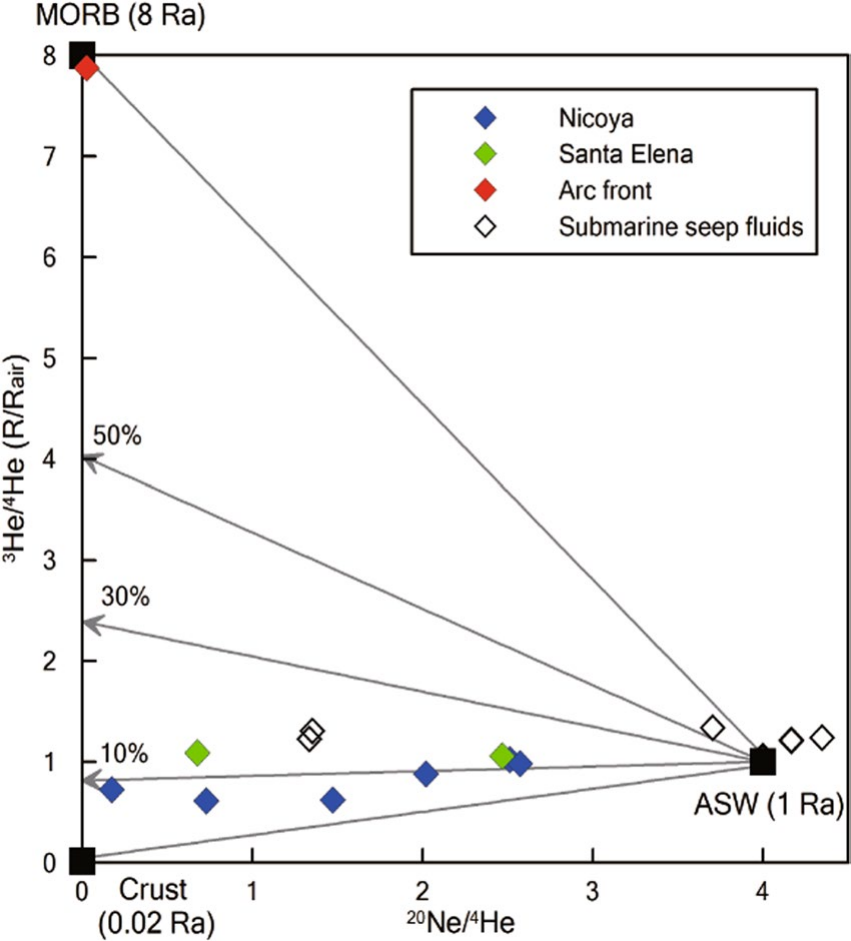
Plot of helium isotope ratios (^3^He/^4^He) versus ^20^Ne/^4^He with three components are MORB, crust, and ASW. The Nicoya and Santa Elene forearc springs show the mixing relationship between ASW and the hybrid fluid with 10% MORB contribution. The arc front spring, located near the Rincon de la Vieja volcano (Fig. 1) is like the MORB component. The submarine seep fluids^8^ are also mixed by two components (ASW and deep fluids with > 10% MORB input).

^3^He/^4^He and ^4^He/^20^Ne ratios of the Nicoya and Santa Elena forearc springs range from 0.61 to 1.09 Ra (Ra is (^3^He/^4^He) air = 1.382 × 10^−6^, ref.^28^) and 0.24 to 5.33, respectively (Table 2). One arc front spring near Rincon de la Vieja volcano (Fig. 1) has ^3^He/^4^He and ^4^He/^20^Ne ratios of 7.88 Ra and 20.0, respectively (Table 1), which is a typical feature of Costa Rican volcanic fluids^5,6^. As ^40^Ar/^36^Ar ratios (296.7 ± 7.5) are close to air (^40^Ar/^36^Ar = 295.5) (Supplementary Information), the ^4^He/^20^Ne ratios of dissolved gases in most of the spring samples are close to the ASW ratio (0.25 at 0 °C, ref.^29^). This implies that the atmospheric contribution is significant for noble gases. In order to linearly extrapolate to the source ^3^He/^4^He ratios of the dissolved gases, we use the ^20^Ne/^4^He ratios (Fig. 5) with the assumption that the source does not contain air-derived ^20^Ne. The extrapolated ^3^He/^4^He ratios fall between the MORB and crustal end members^30–32^. In Fig. 5, all the forearc springs plot on the line indicating 10% mantle helium similar to what has been measured in submarine seep fluids off the coast of Costa Rica^8^, implying that mantle fluids exist in the Nicoya and Santa Elena complexes.

## Discussion

N isotope compositions (δ^15^N = −4.4 to 1.6‰) of all samples collected in Costa Rica (9–11 N°) indicate that lower proportions of N associated with pelagic sediments are released by most of the springs compared to Nicaragua (Fig. 4 and Supplementary Information). Costa Rican volcanic arc gases have a smaller fraction of samples which have δ^15^N values heavier than air (0‰) compared to Nicaragua (Figs 3 and 4)^3,6^. In Fig. 4b and Supplementary Information, the Nicoya forearc springs have less sediment fractions (f_sediment_ = 0–3%) compared to the arc front springs (f_sediment_ = 0–36%) implying that progressive N devolatilization of the subducted slab underneath Costa Rica is occurring. But, the N release from sediment into the Costa Rican arc is significantly less than in the Nicaraguan and Guatemalan arc sections where f_sediment_ is 46–96%^12^ and 20–90%^3^, respectively. There are still outliers on the corrected N provenance diagram, and further studies are required to consider kinetic N isotope fractionation processes, such as denitrifying bacteria activities in forearc areas as proposed by ref.^33^.

Helium isotope ratios (^3^He/^4^He = 0.61–1.09 Ra) of the Nicoya and Santa Elena forearc areas are dominated by a crustal component. Lower ^3^He/^4^He ratios (<2 Ra) are common in other forearc springs, such as Japan, the North Island of New Zealand, and the Kamchatka peninsula of Russia^18^. In Fig. 5, the extrapolated end-member of helium isotope ratios can be determined because deep sources (higher ^4^He/^20^Ne ratios) without severe air contamination can be displayed on the y-intercept. Taking linear mixing lines with different MORB (8 Ra) and crustal (0.02 Ra)^25^ inputs into account, the Nicoya and Santa Elena forearc springs are mainly derived from crustal fluids with significant atmospheric contribution (Fig. 5). It has been known that basement rocks of Nicoya and Santa Elena are uplifted Caribbean large Igneous Province (CLIP) components which formed during Late Cretaceous associated with the Galapagos plume activity^34^. The crustal feature of ^3^He/^4^He ratios in forearcs could be ascribed to old basement rocks resulting in radiogenic ^4^He production by U-Th decay^18^.

There are also other lines of geochemical evidence that indicate weak sediment input in the Costa Rican subduction zone. Ba/La ratios of the Costa Rican lavas (<70) are lower than other Central American margin segments (e.g., Nicaragua, El Salvador, and Guatemala) which are up to ~130 (ref.^35^). Much lower contents of ^10^Be have been reported in the Costa Rican lavas than the Nicaraguan lavas^36^. Pb and Nd radiogenic isotopes imply that magma sources at the Costa Rican volcanic front are less likely affected by sediments^37,38^. Several models have been suggested to account for less sediment contribution in Costa Rica. First, uppermost sediments enriched in organic materials are removed by underplating^39^. This process would result in less N contribution into the arc systems. But, the ODP legs 170 and 205 of off-shore Costa Rica show pelagic sediments are in fact subducting beyond the trench^7^. Second, the shallower slab dip at Costa Rica having warmer thermal regime^40^ would result in N loss at shallow depths through forearc devolatilization as proposed by ref.^41^ based on exhumed metamorphic rocks. This model has been adopted to explain limited fluid availability due to fluids released by metamorphic reactions in the Costa Rican arc^42–44^. The proposed sediment-derived N loss at forearc depths is invalid because forearc springs in Nicoya and Santa Elena have only small sediment contributions (f_sediment_ = 0–3%), though this may be a factor in the southernmost region of the arc (Osa and Burica samples). Finally, it is also unlikely that the incoming plate has a different composition and volume of sediments because off-shore Costa Rica has similar lithology and thickness (400 m) in sediments subducted into the trench (ODP site 1039) to off-shore Guatemala (DSDP site 495)^7,9^.

The subduction erosion model invokes the removal of continental material at the frontal or basal areas of continental margins. At the Costa Rican subduction zone, this model has been advocated by refs^37,45–49^. Compared to the Nicaragua and Guatemalan segments, Costa Rica has abundant seamounts on the Cocos plate at 9–11 N° (Fig. 1)^48^, which could enhance basal subduction erosion to have less signals of pelagic sediments^36,37^. In addition, scars observed in the upper plate in the frontal arc in Costa Rica are caused by seamount subduction colliding with the overriding plate^50^ (Fig. 1).

This model can explain observed N isotope variations in the Costa Rica forearc and arc front. N isotope compositions of the Costa Rican springs at 9–11 N° (δ^15^N = −4.4 to 1.6‰) and reported values of volcanic arc gases (δ^15^N = −3.0 to 1.9‰, refs^3,6,20^) are well consistent with the ranges of low-grade serpentinites (δ^15^N = 0.6 ± 3.4‰) and oceanic crust (δ^15^N = −1.2 ± 3.7‰)^51^. These values are consistent with the observation that the Nicoya and Santa Elena forearc areas are ophiolite complexes at the western edge of the CLIP^52–54^. Although the range of δ^15^N values is close to the MORB value (−5‰), noble gases indicate that the N sources of forearc springs could be crustal (Fig. 5). Hence, the ophiolitic materials which could preserve the MORB-derived N are likely the primary N source in Nicoya and Santa Elena forearc springs. In Fig. 3, most of arc front springs and volcanic gases^3,6^ are slightly heavier than the Nicoya and Santa Elena springs. This slight difference between forearc and arc front springs is likely due to increased δ^15^N values during progressive devolatilization resulting in decrease of ^14^N in the remaining materials^7^. Seamount subduction is not observed at < 9 N° at the Osa and Burica peninsulas consistent with heavier δ^15^N values of the Osa and Burica springs, which is likely attributed to a smaller degree of subduction erosion in this region.

Globally, there are other areas associated with seamount subduction, such as the Sangihe (δ^15^N = −7.3 to 2.1‰, ref.^15^) and Mariana (δ^15^N = −2.5 to 1.6‰, ref.^16^) arcs where ^15^N depleted signatures have been documented. The bathymetry of the Molucca sea floor in front of the Sangihe arc is not as smooth as nearby Celebes sea and Philippine sea plates due to the central ridge^56^. Also, the bathymetric map of the northwest Pacific^57^ shows that the ocean floor is rough with numerous seamounts in front of the Mariana subduction zone. Therefore, the subduction erosion of serpentinized overlying materials (e.g., low-grade serpentinite) enhanced by seamount subduction could result in contribution of N with the ranges of δ^15^N values reported in ref.^51^. Then, N from the upper plate materials could be the source releasing fluids with lighter N isotope compositions, which causes the N mass imbalance at the Costa Rican arc and transports heavier N into the deep mantle as suggested by refs^7,58,59^.

## Conclusions

We report the first N isotopes compositions in the Costa Rican forearc and new N isotopes for arc springs to account for ^15^N-depleted signatures at the Costa Rican arc. Similar to the N isotope compositions reported in volcanic arc gases^3,6,20^, both forearc and arc front springs at 9–11 N° display a similar range of δ^15^N values. In comparison with other tectonic models for the limited amounts of sediment-derived N release (e.g., off-scraping, shallower slab dip, and different lithology and thickness in sediments), the subduction erosion enhanced by seamount subduction at 9–11 N° is a better choice to explain our observations. The δ^15^N values fall within the range of low-grade serpentinite or altered oceanic crust, which is consistent with the observation that the Nicoya and Santa Elena areas have oceanic floor materials formed by the Galapagos plume activity during Late Cretaceous. The seamounts subduction incorporates the overlying plate materials into the arc to release fluids with lighter N isotope values, and progressive devolatilization for N occurs from forearc to arc front. The release of ^15^N-depleted volatiles supports the deep recycling of heavier N at the Costa Rican subduction system.

## Methods

### Sampling and gas geochemistry

The Costa Rican forearc (Nicoya, Santa Elena, Osa, and Burica) and arc front springs were sampled in 2012 and 2014 (Table 1). Spring samples were collected and stored in pre-evacuated Giggenbach bottles, leaving headspaces for gas analyses. Concentrations of gas components (e.g., N_2_, Ar, He, and so on) were obtained in the Volatiles Laboratory at the University of New Mexico (UNM), and the general procedures are described in ref.^60^. He, Ar, O_2_, and N_2_ were measured in dynamic mode on a Pfeiffer Quadrupole Mass Spectrometer (QMS, analytical errors < 1%) with a mass range from 0 to 120 amu and a secondary electron multiplier detector. CO_2_, CH_4_, H_2_, Ar + O_2_, N_2_, and CO contents were determined using a Hayes Sep pre-column and 5 Å molecular sieve columns on a Gow-Mac series G-M 816 Gas Chromatograph (GC, analytical errors < 2%) with a helium carrier gas. A discharge ionization detector was used for CO_2_, CH_4_, H_2_, Ar+O_2_, N_2_ and CO. Concentrations of all gas components were acquired after merging the data from QMS and GC (whole results in Supplementary Information).

### Isotope analyses

Determination of N isotope compositions was conducted using splits of gas samples taken into glass tubes and sealed on high vacuum lines. We neglected the mass interference by carbon monoxide based on its low concentrations (Supplementary Information). Then, N isotope compositions were analyzed on a Thermo Delta V Plus isotope ratio mass spectrometer (IRMS) with a gas bench in the center for Stable Isotopes at UNM. A tube-breaker and a six-way valve were used to break sealed glass tubes and inject N into the IRMS as describe in ref.^61^. Experimental errors (1σ = 0.1‰) for δ^15^N were obtained using multiple measurement of air samples (δ^15^N = 0‰). Argon isotope ratios (^40^Ar/^36^Ar) were determined in static mode on QMS after purification using a cold trap (at liquid N temperature) and hot titanium getters (at 550 °C) in the Volatiles Laboratory at UNM. Cu tubes were used for helium isotope analyses because helium can penetrate the glass containers (e.g., Giggenbach bottle). ^3^He/^4^He ratios were acquired by a Helix-SFT noble gas mass spectrometer at the Atmosphere and Ocean Research Institute of the University of Tokyo (AORI). He and Ne were purified using hot titanium getters (held at 400 °C) and charcoal traps (at liquid N temperature) and ^4^He/^20^Ne ratios were obtained by an on-line QMS. After that, neon was trapped using a cryogenic trap (at 40°K). Experimental errors (1σ) for and ^3^He/^4^He and ^4^He/^20^Ne ratios are about 1% and 5%^62^.

## Electronic supplementary material

Dataset 1

Dataset 2
